# Modulatory effects of isoflavones and zearalenone metabolites on deoxynivalenol induced gut barrier toxicity *in vitro*

**DOI:** 10.1007/s12550-026-00645-1

**Published:** 2026-03-30

**Authors:** Dino Grgic, Barbara Novak, Elisabeth Varga, Doris Marko

**Affiliations:** 1 Department of Food Chemistry and Toxicology, Faculty of Chemistry, University of Vienna, Währinger Str. 38-40, Vienna, 1090 Austria; 2 Doctoral School in Chemistry, University of Vienna, Währinger Str. 38-42, Vienna, 1090 Austria; 3 dsm-firmenich, Animal Nutrition & Health R&D Center, Technopark 1, Tulln, 3430 Austria; 4 Food Hygiene and Technology, Centre for Food Science, Clinical Department for Farm Animals and Food System Transformation, University of Veterinary Medicine, Veterinärplatz 1, Vienna, Vienna 1210 Austria

**Keywords:** Daidzein, Genistein, Decarboxylated hydrolyzed zearalenone, Intestine, Permeability, IPEC-J2

## Abstract

Humans and animals are frequently exposed to dietary compounds that can adversely affect intestinal health. The *Fusarium* mycotoxin deoxynivalenol (DON) is known to compromise epithelial barrier integrity. DON frequently co-occurs in food and feed with the mycoestrogen zearalenone (ZEN) and soy-derived isoflavones (ISFs) such as genistein (GEN) and daidzein. Therefore, this study investigates the effects of ZEN (10 nM), its metabolites hydrolyzed ZEN (HZEN) and decarboxylated hydrolyzed ZEN (DHZEN) (each 10 nM), and ISFs (GEN and equol (EQ), 10 µM), alone and in combination with DON (10 µM), on intestinal barrier function using the porcine epithelial cell line IPEC-J2. Barrier integrity was assessed via transepithelial electrical resistance (TEER) and paracellular permeability (lucifer yellow). DON (10 µM) significantly reduced barrier integrity, confirming its disruptive effect. Conversely, treatment with ZEN, HZEN, DHZEN, GEN, and EQ did not impair barrier function. Co-exposure of DON with ZEN or HZEN yielded TEER values similar to those observed with DON alone. In contrast, the DHZEN + DON combination resulted in higher TEER values compared to DON alone, suggesting a mitigating effect under the experimental conditions. Quantification of GEN by LC-MS/MS showed consistent levels across treatments, except for reduced basolateral GEN during co-incubation with DHZEN, suggesting altered transport or metabolism. These findings highlight the ability of DHZEN to mitigate the intestinal barrier disruption induced by DON and underscores the complex interaction of maintaining epithelial barrier integrities.

**Supplementary Information:**

The online version contains supplementary material available at 10.1007/s12550-026-00645-1.

## Introduction

**Fig. 1:**
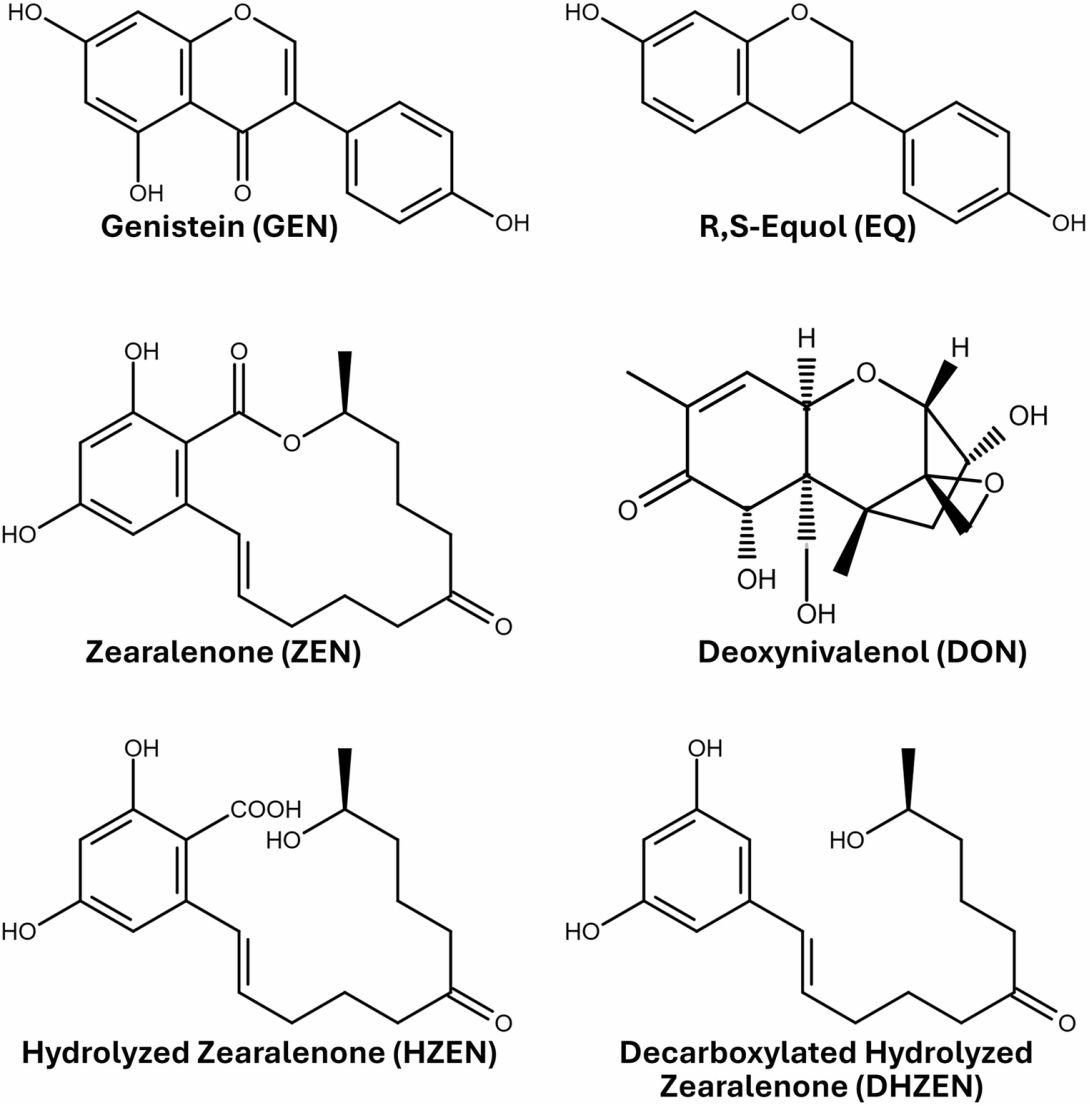
Chemical structures of the investigated isoflavone genistein, as well as the gut metabolite of daidzein R,S-equol (EQ) and mycotoxins (zearalenone, deoxynivalenol, hydrolyzed zearalenone and decarboxylated hydrolyzed zearalenone) (created with: chemspace.com)

The use of soy-based products as feed components and plant-based alternatives to meat has gained significant popularity, primarily due to their high protein content and potential health benefits associated with isoflavones (ISFs), such as genistein (GEN), daidzein (DAI), and its gut microbial metabolite R, S-equol (EQ) (Fig. 1) (Qin et al. 2022). Epidemiological studies in humans have linked long-term intake of ISFs to various positive health effects. In Asian countries where soy is a common dietary component, the incidence of breast, prostate, and colorectal cancer is lower compared to regions with lower soy consumption (Jou et al. 2008; Wu et al. 2008; Yan and Spitznagel 2009). Moreover, ISFs have been associated with the prevention of coronary heart disease, osteoporosis, and decline of cognitive function (Sacks et al. 2006; Sarkar and Li 2003). In animals, beneficial properties attributed to ISFs include growth promotion, enhanced antioxidative and immune function (Grgic et al. 2021). However, high intake of ISFs may also lead to adverse health effects, particularly affecting the reproductive tract of female farm animals (Bennetts et al. 1946; Hashem and Soltan 2016). Reported effects include infertility, uterine prolapse, and swelling of the mammary glands and vulva (Grgic et al. 2021). Additionally, the Scientific Committee on Consumer Safety has restricted GEN to 0.007% and DAI to 0.02% in cosmetic products due to concerns regarding their potential endocrine-disrupting properties (European Commission 2024).

The presence of high ISF concentrations, in combination with the mycotoxins zearalenone (ZEN) and deoxynivalenol (DON), in finished feed samples raises concerns about the potential combinatory toxic effects (Grgic et al. 2021; Penagos-Tabares et al. 2021, 2023). ISFs and their glycosides were present at significant median level in pig feed. In the same commodity, ZEN and DON were also found in higher concentrations. These findings suggest a frequent co-occurrence of these phytoestrogens and mycotoxins and thus, the need to investigate possible effects in combinations.

An evaluation by the European Food Safety Authority (EFSA) in 2011 concluded that chronic human exposure to ZEN remains within safe limits. Nevertheless, concerns persist due to the extensive use of corn and soybean-based products in human diets and livestock feed formulations (EFSA 2011). The reproductive toxicity of ZEN in both humans and animals is well documented (Kowalska et al. 2016; Minervini and Aquila 2008). The phase I metabolite α-zearalenol (α-ZEL) exhibits even higher estrogenic potency than its parent compound, highlighting the need for effective methods to degrade ZEN in feed (Frizzell et al. 2011; Grgic et al. 2022). Estrogenic signaling has been shown to play a regulatory role in intestinal barrier integrity, as the endogenous estrogen 17β-estradiol (E2) upregulates tight junction protein expression and enhances barrier function via estrogen receptor–mediated pathways (Choi et al. 2018). The enzyme zearalenone hydrolase (ZENase, EC 3.1.1.-), marketed as ZEN*zyme*^®^ by BIOMIN Holding GmbH, Getzersdorf, Austria, has been shown to catalyze the conversion of ZEN to hydrolyzed ZEN (HZEN, Fig. 1) and decarboxylated hydrolyzed ZEN (DHZEN, Fig. 1). This results in a significant reduction in estrogenic activity of the hydrolyzed metabolites, as demonstrated primarily in mechanistic in vitro assays and supported by in vivo data obtained under experimental high-dose conditions (Fruhauf et al. 2019). As zearalenone exhibits a systemic bioavailability of 78–87% in pigs, maintaining an intact intestinal barrier is critical to limit toxin translocation and subsequent adverse effects (Dänicke and Winkler 2015). DON, on the other hand, has been associated to impair intestinal barrier integrity (Akbari et al. 2017). A consistent decrease in transepithelial electrical resistance (TEER) has been observed in the presence of DON, indicating a disruption of tight junctions (TJ) and, consequently, alterations to the integrity of the intestinal epithelium. These changes also affect specific TJ proteins, such as claudin-4 (CLD-4), whose expression is decreased upon exposure to DON (Pinton et al. 2009). Moreover, DON has been shown to impair nutrient absorption and induce signs of intestinal inflammation (Maresca et al. 2008). As the intestine acts as a selective barrier for nutrient exchange and first line of defense against contaminants, any impairment of this barrier increases the epithelial permeability, allowing potentially harmful compounds to pass through (Abia et al. 2017). To investigate the effects of these mycotoxins in combination with ISFs on intestinal barrier functions, the porcine intestinal epithelial cell line IPEC-J2 was employed. The impact of co-occurring phytoestrogens (GEN, EQ) and mycotoxins (DON, ZEN, HZEN, DHZEN) on barrier integrity was evaluated using TEER measurements, followed by assessment of paracellular permeability with lucifer yellow (LY). Considering the well-documented capacity of DON to disrupt intestinal integrity, the primary objective of this study was to assess potential synergistic or antagonistic effects, as well as metabolite changes resulting from combined exposures.

## Materials & methods

### Cell culture

The non-transformed jejunal intestinal porcine epithelial cell line known as IPEC-J2 (ACC 701; Leibnitz Institute DSMZ, German Collection of Microorganisms and Cell Cultures, Braunschweig, Germany) (passage number 8–13) was used to perform cell culture experiments. Cells were cultured in Dulbecco’s modified eagle medium (DMEM/F12 in a 1:1 ratio, PAN-Biotech, Aidenbach, Germany), supplemented with 5% fetal bovine serum, 1% insulin-transferrin-selenium, 5 ng/mL epidermal growth factor, 5 mM Glutamax (all sourced from Gibco™, Life Technologies, Carlsbad, CA/USA), and 16 mM 4-(2-hydroxyethyl)−1-piperazineethanesulfonic acid (Sigma-Aldrich, St. Louis/MO, USA). Cultivation was performed at 37 °C and 5% CO₂ under controlled conditions until cells reached approximately 80% confluency.

Cultivation practices involved seeding cells at a concentration of 1 × 10^6^ cells/mL in 175 cm^2^ tissue culture flasks (Sarstedt, Nümbrecht, Germany) with 32 mL of complete cultivation medium. Cells were grown to confluence and then, detached for further experiments using a solution of trypsin (0.25%) and ethylenediaminetetraacetic acid (EDTA, 0.5 mM), ensuring efficient harvesting for further analysis.

### Chemicals

Phosphate-buffered saline (PBS) (10x) was prepared by dissolving 1.71 M of NaCl, 100 mM of Na_2_HPO_4_, 34 mM of KCl and 18 mM of KH_2_PO_4_ in autoclaved water. After adjusting the pH-value to 7.4, the solution was sterile filtered and stored at 4 °C until further usage. PBS (1x) was prepared by diluting PBS (10x) 1:10 with distilled water. DON (from *Fusarium* sp., ≥ 95%, Biopure, RomerLabs^®^, Tulln, Austria) was dissolved in sterile autoclaved water to a stock concentration of 6.75 mM and was further diluted to the desired working concentrations in complete cultivation medium. ZEN, LY and neutral red (NR) were obtained from Sigma Aldrich Chemie GmbH (Schnelldorf, Germany). HZEN and DHZEN were produced in-house by dsm-firmenich according to their standard operating procedure using the enzyme zearalenone lactonase Zhd101p (Fruhauf et al. 2019; Vekiru et al. 2016). R, S-equol (99.3%) and GEN (> 99.9%) were acquired from LC Laboratories (Woburn/MA, USA). (R, S)-equol-4’-sulfate (E4’S) (90%) and (R, S)-equol-7-glucuronide (E7G) (98%) were obtained from Santa Cruz Biotechnology Inc. (Dallas/TX, USA). Genistein-4’,7-disulfate (G4’,7dS) (99.4%) was synthesized from Max Rubner Institute (Karlsruhe, Germany) as described by Soukup et al. (2014). All other phase II metabolites (genistein-4’,7-diglucuronide (G4’,7dG) (90.8%), genistein-4’-glucuronide (G4’G) (99%), genistein-4’-glucuronide-7-sulfate (G4’G7S) (98.9%), genistein-7-glucuronide (G7G) (95%), genistein-7-glucuronide-4’-sulfate (G7G4’S) (99%) and genistein-7-sulfate (G7S) were acquired from Toronto Research Chemical Inc. (Toronto/ON, Canada). Stock solutions of ZEN, HZEN, and DHZEN (20 µM) and ISFs (20 mM) were prepared in dimethyl sulfoxide (DMSO), while DON stock solution (20 mM) was prepared in sterile water.

### Dose selection

The doses used in this study were selected based on a combination of reported occurrence data for ISFs and ZEN in pig feed (approximately 1,000:1) (Grgic et al. 2021), biological relevance, technical feasibility in the IPEC-J2 model, and the need to elicit measurable cellular responses without inducing excessive cytotoxicity.

DON, GEN, and EQ were tested at 10 µM, whereas ZEN was applied at 10 nM. The DON concentration of 10 µM was chosen because it is close to the threshold of mild cytotoxicity in IPEC-J2 cells, allowing the investigation of stress- and barrier-related effects without causing extensive cell death. Higher DON concentrations were not feasible due to pronounced cytotoxicity in this cell model. At this concentration, DON is able to disrupt intestinal barrier integrity and was therefore used as a barrier-disrupting agent.

In contrast, ZEN was tested at 10 nM, reflecting its high estrogenic potency and its ability to elicit biological effects at nanomolar levels. In comparison with ZEN, both GEN and EQ exhibit significantly reduced endocrine-activity. Consequently, the concentrations of these compounds were selected to achieve comparable biological activity, rather than equimolarity with ZEN. E2 has been demonstrated to upregulate tight junction proteins and enhance barrier function via estrogen receptor signaling (Choi et al. 2018). In accordance with this evidence, ISFs and ZEN were applied at concentrations reflecting their maximal estrogenic potential.

### Measurement of transepithelial electrical resistance (TEER)

All experiments were repeated in at least four independent biological experiments performed on separate days with independently prepared IPEC-J2 cells. Each biological replicate included duplicate wells per treatment condition (technical replicates). For statistical evaluation, the mean of the technical replicates was calculated for each independent experiment and used as one biological replicate. IPEC-J2 cells were seeded in the apical compartment of specialized inserts from Sarstedt (12-well, polyethylene terephthalate (PET), membrane pore size: 0.4 μm, cell growth area: 1.1 cm^2^, membrane thickness: 12 μm, nominal pore density: 2 × 10^6^/cm^2^) at a density of 1 × 10^5^ cells per insert and allowed to differentiate upon reaching confluency for a period of 8 days. After the differentiation phase, cells were exposed apically to various treatments of ZEN, HZEN, DHZEN (all 10 nM), GEN and EQ (both 10 µM) as single substances or in the respective combinations with and without 10 µM DON in culture medium throughout the experimental period (24–72 h). The substances were previously dissolved in 2000 times higher concentrations, followed by dilution in the assay medium. In case of single substance testing and for the solvent control, DMSO was added to reach 0.1% in the final incubation solutions. To assess the integrity of the cell layers, TEER measurements were performed prior incubation (0 h) as well as after 24, 48, and 72 h of incubation using the Millicell-Electrical Resistance System (ERS, Merck Millipore, Billerica/MA, USA). To ensure the reliability of results, the culture plates were carefully placed on a plate heater set at 37 °C to avoid temperature fluctuations prior to the TEER measurements. Furthermore, the chopstick electrodes were rinsed with ethanol and allowed to equilibrate in culture medium at room temperature. TEER values obtained from each well were multiplied by the corresponding membrane area. To establish a baseline, the TEER value of a blank insert containing only culture medium (without cells) was subtracted from the values of the treated wells. Results were then presented as a percentage relative to the solvent control, providing a standardized and reliable comparison of the results.

### Measurement of paracellular permeability by lucifer yellow

Following the final TEER measurements, the small hydrophilic compound lucifer yellow (LY) was used to assess the integrity of the cellular monolayers and detect changes in the paracellular permeability in treated cells. Before LY application, both compartments were rinsed with Hank’s balanced salt solution (HBSS) buffer, containing D-glucose, HEPES, CaCl_2_, and MgCl_2_ adjusted to a pH value of 7.4.

Subsequently, 0.5 mL of 0.1 mg/mL LY CH dilithium salt in HBSS buffer was added to the apical compartment, while 1.5 mL HBSS buffer was added to the basolateral compartment. Cell plates were then incubated at 37 °C and 5% CO_2_ for one hour. After incubation, the fluorescence of the basolateral medium and the pure LY solution were measured in triplicates using excitation wavelength of 485 nm and emission wavelengths of 535 nm with a Cytation 3 Cell Imaging Multi-Mode Reader from Biotek^®^ (Winooski/VM, USA).

To quantify the results, the fluorescence values of the sample wells were normalized to the fluorescence of 0.1 mg/mL LY, subtracting the blank (only HBSS) fluorescence (Groestlinger et al. 2022; Beisl et al. 2021). Data are provided as a percentage, showing the extent of paracellular passage of LY through the cellular monolayers. In our experimental setup, to classify monolayers as tightly sealed, a LY permeability of less than 1% was considered acceptable in the solvent control. Four biological replicates, each consisting of two technical replicates, were prepared to ensure reproducibility of the results.

### Measurement of cell viability by neutral red assay

After the LY measurement, cell viability was examined via the NR assay. Following the 72 h incubation and the one-hour LY treatment, all buffers were removed, and cells were washed with PBS. Following, a NR solution (40 µg NR/mL medium) was added with a final dilution of 1:100 in complete cultivation medium at 37 °C for 3 h. Afterwards, cells were washed with PBS and 350 µL of a destaining solution (50% absolute ethanol, distilled H_2_O and 1% glacial acetic acid) were added to each insert and incubated for 10 min on a plate shaker. Next, 100 µL of each insert was transferred to individual wells of a fresh 96-well plate. The quantity of dye incorporated into cells was measured photometrically at a wavelength of 540 nm with a reference wavelength of 690 nm using a microplate reader (Biotek, Winoosky/VT, USA). The measured absorbance is directly proportional to the number of viable cells. Viability was calculated as percentage normalized to the solvent control, which was set to 100%.

### High performance liquid chromatography coupled to tandem mass spectrometry (HPLC-MS/MS)

After 72 h incubation in the TEER method, 80 µL supernatant was transferred to microtubes containing 80 µL ice cold acetonitrile (ACN), resulting in a 1:2 dilution, and stored at −80 °C until further processing. Solutions were thawed, centrifuged (10 min at 4 °C and 18,620 rcf) and 100 µL of supernatant was transferred to HPLC vials with micro-inserts (both Bruckner Analysentechnik GmbH, Linz, Austria) for measurement and 2 µL of the sample were injected into the hyphenated HPLC-MS/MS system. Samples were analyzed using a 1260 Infinity II LC System (Agilent, Santa Clara/CA, USA) coupled to a QTRAP^®^ 6500+ from Sciex (Framingham/MA, USA). The developed high-performance liquid chromatographic tandem mass spectrometric (HPLC-MS/MS) method was based on Soukup et al. (2014). Separation was performed on a Supelco Ascentis^®^ Express C18 column (100 × 2.1 mm, 2.7 μm) and a C18 guard column (Phenomenex, Aschaffenburg, Germany) with 10 mM ammonium carbonate in water and ACN/methanol (1/2.5, *v/v*) as eluents A and B. The autosampler temperature was set to 5 °C, and the column temperature was 40 °C. The flow rate was 0.5 mL/min and the applied gradient was as follows: a 2.6 min holding period at 3% B was followed by a linear increase to 56% until 10.7 min and steep increase to 95% until 11 min. Thereafter, the column was flushed with 95% for three minutes before the column was equilibrated at 3% B until the end of the method at 16.5 min. Data were acquired in multiple reaction monitoring (MRM) mode using negative electrospray ionization and specific transitions are provided in Supplement Table S1. All compounds were previously optimized using syringe injection of ACN-stock solutions (100 mg/L) diluted with eluent A: B (1:1, *v/v*). Qualitative data evaluation was performed with Analyst (v.1.7.) with HotFix 3. External calibration standards were prepared from individual stock solutions (100 mg/L in ACN) by first preparing a master-mix which was further diluted in serial dilutions with (MeOH: H_2_O, 30:70) to obtain concentration between 0.1 and 3000 µg/L. Quantitative analytes were performed with Skyline (v. 22.2; MacCoss Lab, Department of Genome Sciences, University of Washington, Seattle/WA, USA).

### Statistics

TEER measurements, LY and NR assays were conducted with technical duplicates in four independent biological replicates (*n* ≥ 4). Outliers were identified using the Nalimov and Kolmogorov-Smirnov tests for normality and excluded from the calculation of mean values and standard deviations. Origin Pro^®^ 2021 software was used for statistical analyses and data plotting with significance levels of α = 0.05, 0.01, and 0.001. Significant differences between the solvent control and the incubation conditions were calculated by one-way analysis of variance (ANOVA) and indicated with “*” (*p* < 0.05), “**” (*p* < 0.01) or “***” (*p* < 0.001). Significant differences between 10 µM DON and the incubation conditions were calculated by two-sample Student’s *t*-test and indicated with “#” (*p* < 0.05), “##” (*p* < 0.01) or “###” (*p* < 0.001). Viability results were assessed using one-way Student’s *t*-tests.

## Results

### Assessment of single substances in the absence and presence of DON on the transepithelial electrical resistance (TEER)

**Fig. 2:**
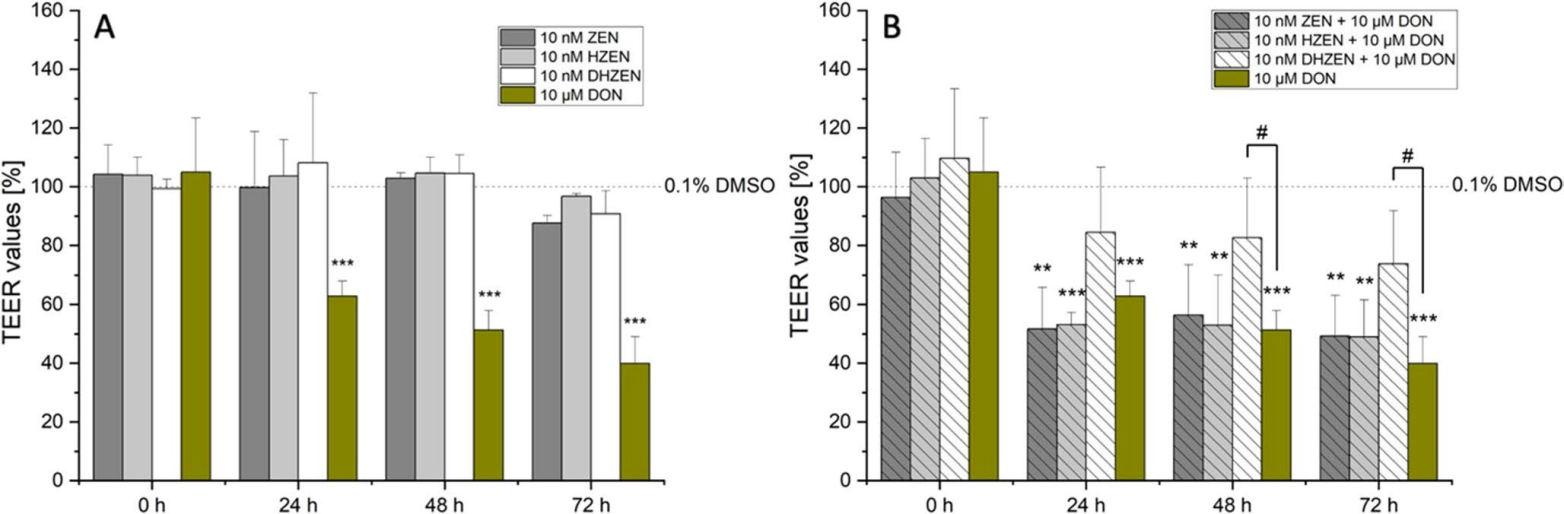
Transepithelial electrical resistance (TEER) of IPEC-J2 cells after 0, 24, 48 and 72 h incubation with (A) zearalenone (ZEN), hydrolyzed ZEN (HZEN), decarboxylated hydrolyzed ZEN (DHZEN), deoxynivalenol (DON) as single substances and (B) DON alone or in combination with ZEN, HZEN or DHZEN. The solvent control is shown as the dashed line (0.1% DMSO). Results are depicted as mean + standard deviation of at least 4 biological replicates and are normalized to the solvent control. Significant differences between the solvent control and the incubation conditions were calculated by one-way analysis of variance (ANOVA) and indicated with “*” (*p* < 0.05), “**” (*p* < 0.01) or “***” (*p* < 0.001). Significant differences between 10 µM DON and the incubation conditions were calculated by two-sample Student’s *t*-test and indicated with “#” (*p* < 0.05), “##” (*p* < 0.01) or “###” (*p* < 0.001)

Exposure of the confluent IPEC-J2 cells to 10 nM ZEN did not significantly alter the TEER values after 24, 48 and 72 h of incubation. The degradation products of ZEN, namely HZEN and DHZEN in concentrations of 10 nM, did not lead to a significant decrease in TEER values. Even after 72 h, TEER values remained relatively high, with mean values for HZEN of 97% (± 1%) and DHZEN of 91% (± 8%), suggesting no impact on barrier integrity. In contrast, and as expected, incubation with 10 µM DON significantly lowered the TEER values after 24 h to an average value of 63% (± 5%), which further decreased after 48 h and 72 h to approximately 51% (± 7%) and 40% (± 9%), respectively (Fig. 2A). When DON was combined with ZEN or HZEN, there were no significant changes compared to DON alone. However, co-incubation with DON and DHZEN showed a counteracting impact on TEER values compared to DON alone with mean TEER values of 88% (± 20%), 87% (± 19%) and 78% (± 17%) after 24, 48 and 72 h (Fig. 2B). Direct comparison between 10 µM DON alone and the combination of 10 nM DHZEN + 10 µM DON show significant differences on TEER values after 48 and 72 h.

**Fig. 3:**
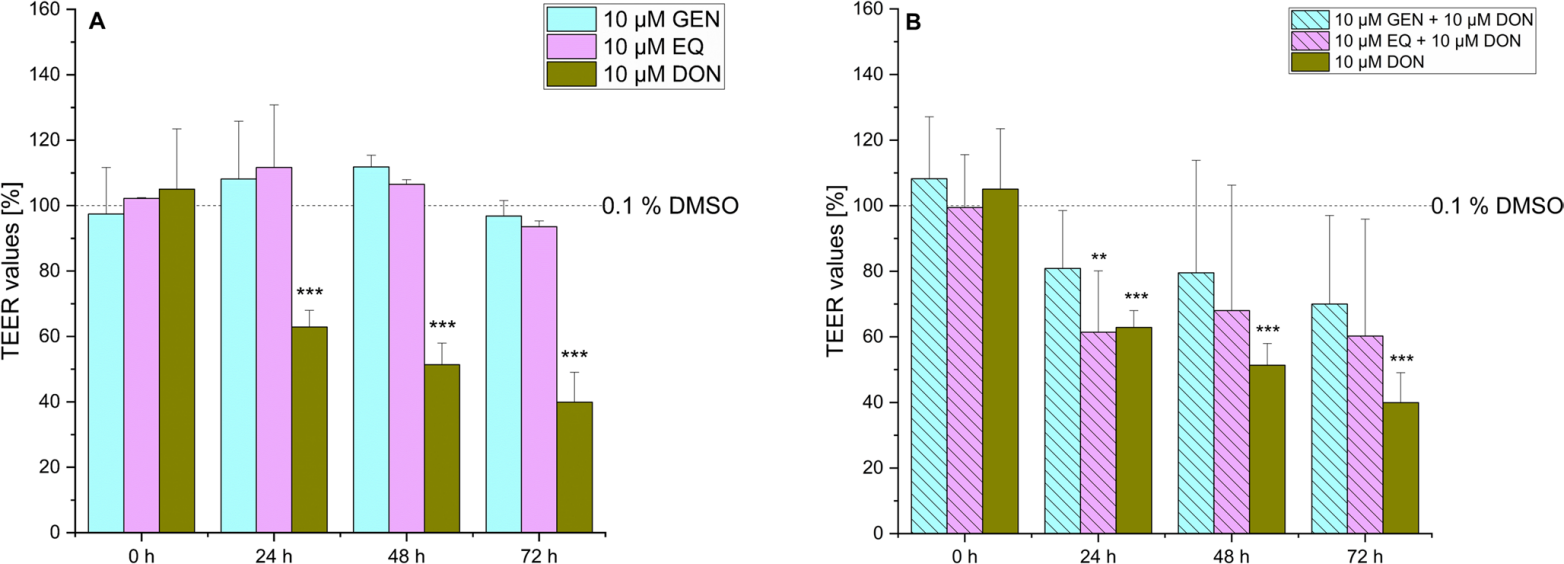
Transepithelial electrical resistance (TEER) of IPEC-J2 after 0, 24, 48 and 72 h incubation with (**A**) genistein (GEN) and R, S-equol (EQ) as single substances or (**B**) deoxynivalenol (DON) alone or in combination with GEN or EQ. The solvent control is shown as the dashed line (0.1% DMSO). Results are depicted as mean + standard deviation of at least 4 biological replicates and are normalized to the solvent control. Significant differences between the solvent control and the incubation conditions were calculated by one-way analysis of variance (ANOVA) and indicated with “*” (*p* < 0.05), “**” (*p* < 0.01) or “***” (*p* < 0.001). Significant differences between 10 µM DON and the incubation conditions were calculated by two-sample Student’s *t*-test and indicated with “#” (*p* < 0.05), “##” (*p* < 0.01) or “###” (*p* < 0.001)

GEN and EQ led to mean TEER values of 108% (± 18%) and 112% (± 4%) for GEN, and 112% (± 19%) and 107% (± 1%) for EQ, respectively. After 72 h, the TEER values for both GEN and EQ returned to the baseline levels comparable to the solvent control (Fig. 3A). When ISFs were co-incubated with 10 µM DON, a significant decrease in TEER values was observed. Specifically, in the presence of 10 µM GEN + 10 µM DON, TEER values decreased to mean values of 81% (± 17%), 80% (± 25%), and 70% (± 22%) after 24, 48, and 72 h, respectively. However, these results were not statistically significant. In the case of EQ + DON, mean TEER values dropped to 61% (± 18%), 67% (± 27%), and 60% (± 24%) over the same time frame. Interestingly, it appears that ISFs have the tendency to exert a protective effect, mitigating the sharp decline in TEER values induced by 10 µM DON alone.

### Assessment of tertiary combinations on the transepithelial electrical resistance (TEER)

**Fig. 4:**
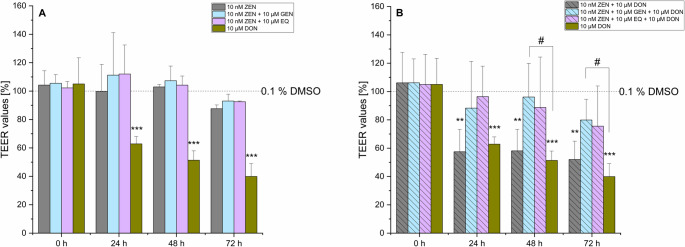
Transepithelial electrical resistance (TEER) of IPEC-J2 after 0, 24, 48 and 72 h incubation with (**A**) zearalenone (ZEN) ± genistein (GEN) and R, S-equol (EQ) or (**B**) a combination of ZEN with deoxynivalenol (DON) ± GEN or EQ. Solvent control is shown as the dashed line (0.1% DMSO). Results are depicted as mean + standard deviation of at least 4 biological replicates and are normalized to the solvent control. Significant differences between the solvent control and the incubation conditions were calculated by one-way analysis of variance (ANOVA) and indicated with “*” (*p* < 0.05), “**” (*p* < 0.01) or “***” (*p* < 0.001). Significant differences between 10 µM DON and the incubation conditions were calculated by two-sample Student’s *t*-test and indicated with “#” (*p* < 0.05), “##” (*p* < 0.01) or “###” (*p* < 0.001)

**Fig. 5:**
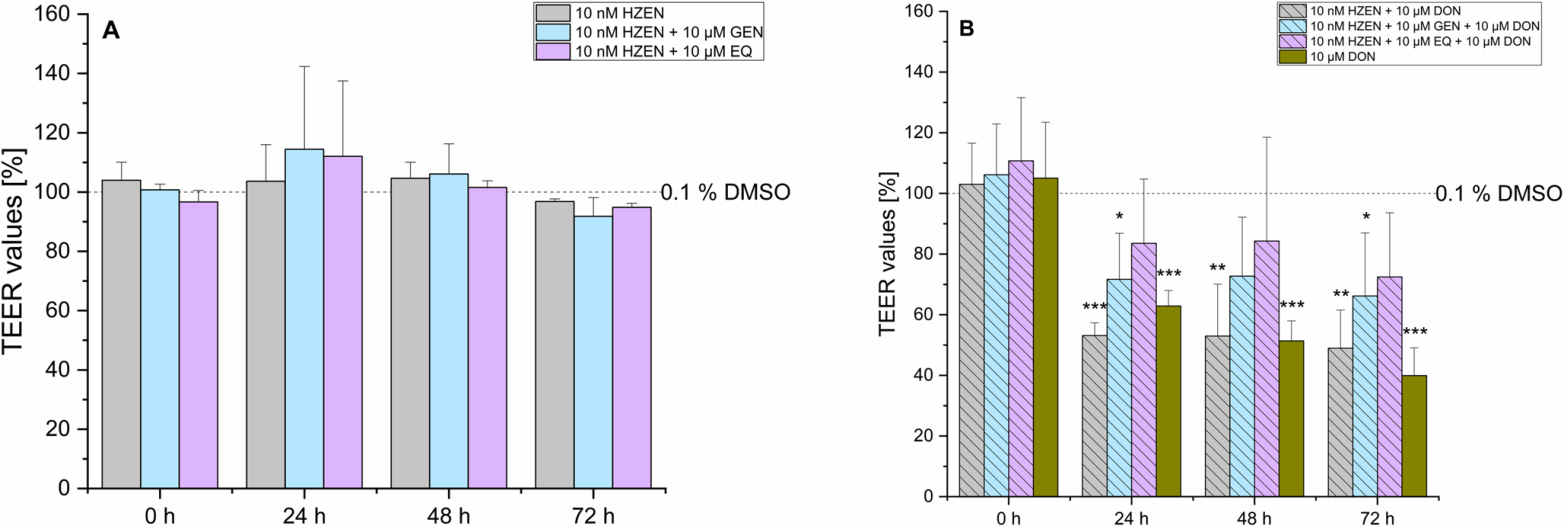
Transepithelial electrical resistance (TEER) of IPEC-J2 after 0, 24, 48 and 72 h incubation with (**A**) hydrolyzed zearalenone (HZEN) ± genistein (GEN) and R, S-equol (EQ) or (**B**) a combination of HZEN with deoxynivalenol (DON) ± GEN or EQ. Solvent control is shown as the dashed line (0.1% DMSO). Results are depicted as mean + standard deviation of at least 4 biological replicates and are normalized to the solvent control. Significant differences between the solvent control and the incubation conditions were calculated by one-way analysis of variance (ANOVA) and indicated with “*” (*p* < 0.05), “**” (*p* < 0.01) or “***” (*p* < 0.001). Significant differences between 10 µM DON and the incubation conditions were calculated by two-sample Student’s *t*-test and indicated with “#” (*p* < 0.05), “##” (*p* < 0.01) or “###” (*p* < 0.001)

**Fig. 6:**
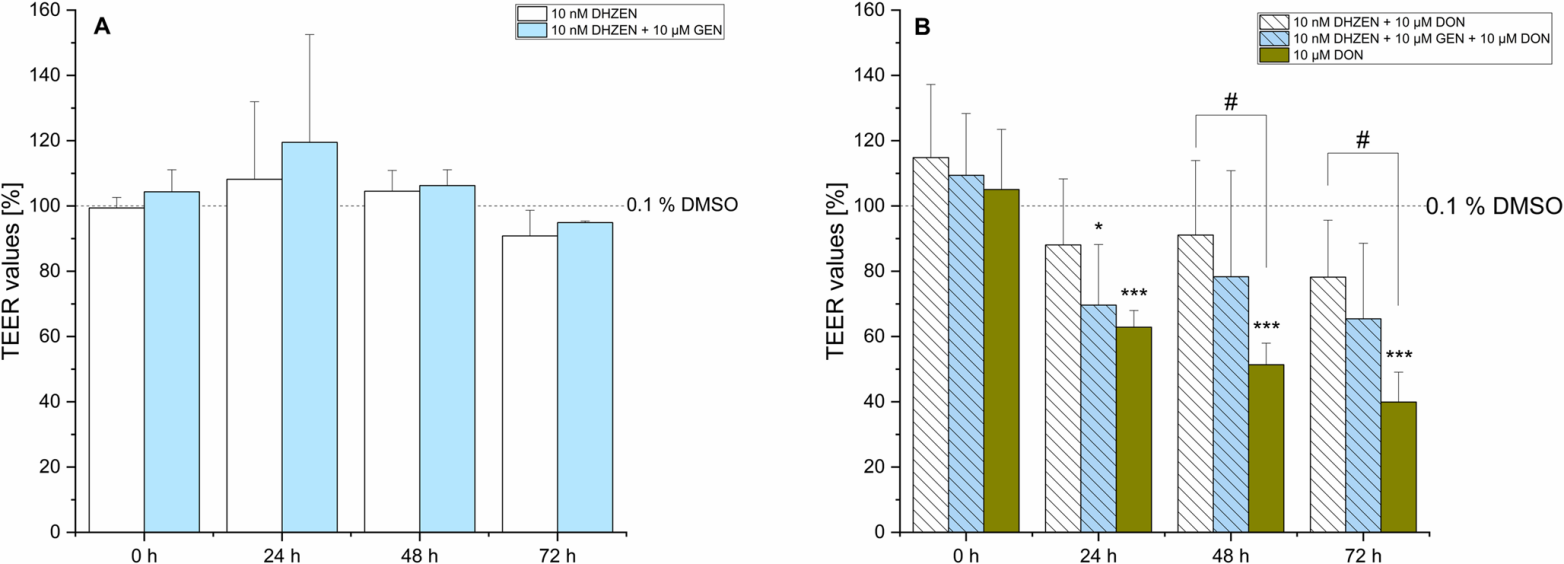
Transepithelial electrical resistance (TEER) of IPEC-J2 after 0, 24, 48 and 72 h incubation with (**A**) decarboxylated hydrolyzed zearalenone (DHZEN) ± genistein (GEN) and R, S-equol (EQ) or (**B**) a combination of DHZEN with deoxynivalenol (DON) ± GEN or EQ. Solvent control is shown in the dashed line (0.1% DMSO). Results are depicted as mean + standard deviation of at least 4 biological replicates and are normalized to the solvent control. Significant differences between the solvent control and the incubation conditions were calculated by one-way analysis of variance (ANOVA) and indicated with “*” (*p* < 0.05), “**” (*p* < 0.01) or “***” (*p* < 0.001). Significant differences between 10 µM DON and the incubation conditions were calculated by two-sample Student’s *t*-test and indicated with “#” (*p* < 0.05), “##” (*p* < 0.01) or “###” (*p* < 0.001)

In combinations of ZEN or its degradation products HZEN and DHZEN with ISFs, a slight but not significant trend towards increased TEER values was observed after 24 h. However, no differences compared to the solvent control were detected after 48 and 72 h (Figs. 4A, 5A and 6A). The addition of ISFs to combinations of ZEN or HZEN with 10 µM DON showed a protective effect by preventing a significant TEER reduction compared to the combination of ZEN or HZEN and 10 µM DON alone (Figs. 4B and 5B). DON alone significantly decreased TEER values compared to the combination of ZEN + GEN + DON. Notably, the combination of DHZEN and DON resulted in mean TEER values of 88% (± 20%), 91% (± 19%), and 78% (± 17%) after 24, 48, and 72 h, respectively, which were not significantly different from the solvent control (Fig. 6B). However, when directly compared with DON alone, the DHZEN + DON combination showed significantly higher TEER values, suggesting an attenuation of the DON-induced barrier-disrupting effect under the experimental conditions applied.

### Permeability assessment by Lucifer Yellow (LY)

**Fig. 7:**
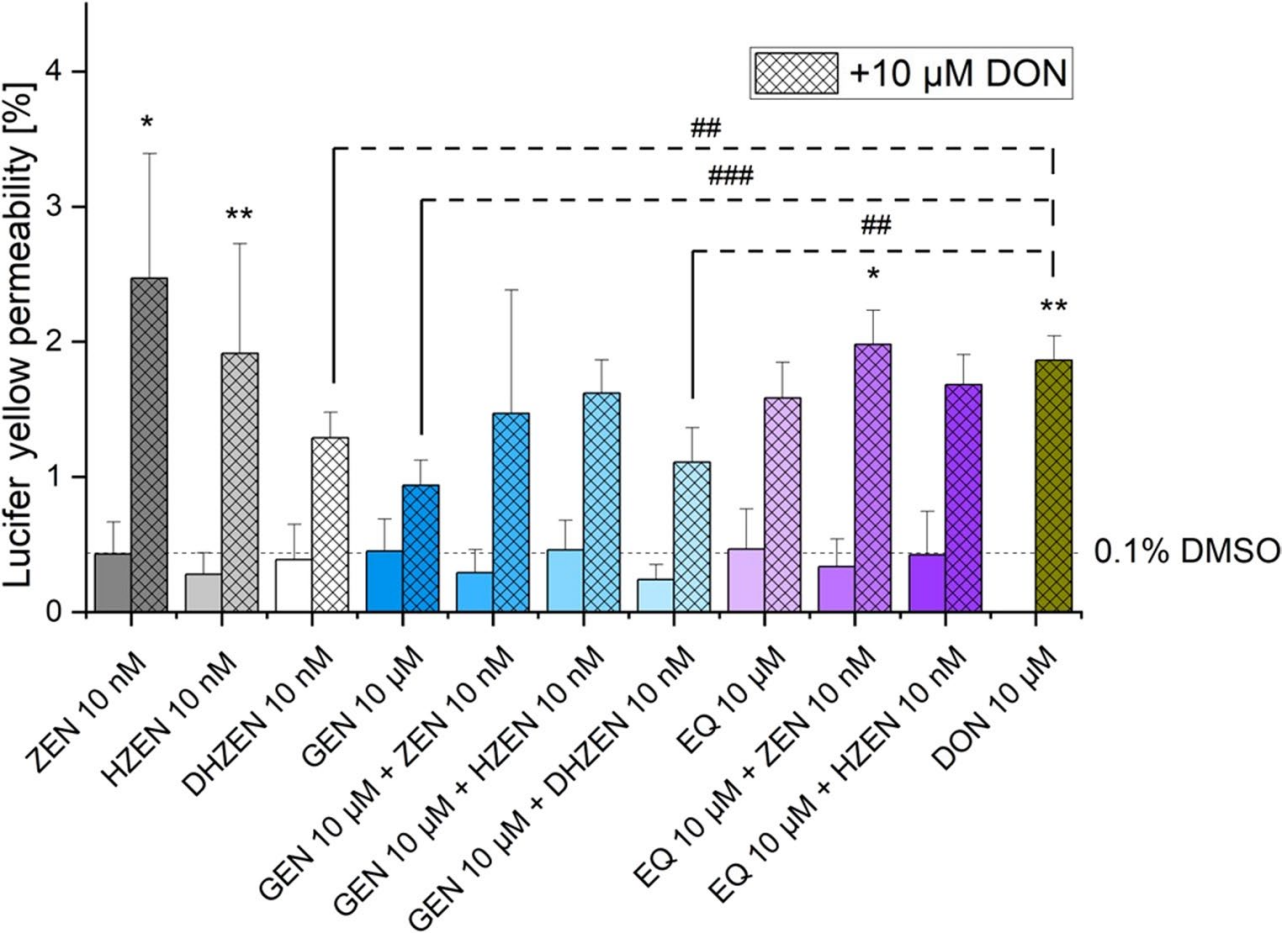
Lucifer yellow (LY) fluorescence intensity in the basolateral compartment after 72 h incubation with deoxynivalenol (DON), zearalenone (ZEN), hydrolyzed ZEN (HZEN), decarboxylated hydrolyzed ZEN (DHZEN), genistein (GEN), GEN + ZEN, GEN + HZEN, GEN + DHZEN, R,S-equol (EQ), EQ + ZEN, EQ + HZEN ± 10 µM DON or solvent control (0.1% DMSO). Results are depicted as mean + standard deviation of 4 biological replicates. Significant differences between the solvent control and the incubation conditions were calculated by one-way analysis of variance (ANOVA) and indicated with “*” (*p* < 0.05), “**” (*p* < 0.01) or “***” (*p* < 0.001). Significant differences between 10 µM DON and the incubation conditions were calculated by two-sample Student’s *t*-test and indicated with “#” (*p* < 0.05), “##” (*p* < 0.01) or “###” (*p* < 0.001)

In our experiments, ZEN, its degradation products HZEN and DHZEN and ISFs without 10 µM DON showed no significant impact on LY permeability when compared to the solvent control (0.1% DMSO). A significant increase in LY permeability was induced by 10 µM DON which was 1.8% ± 0.2%. Combinations of 10 µM DON with either ZEN (2.4% ± 0.9%) or HZEN (1.9% ± 0.8%) resulted in similar LY permeability levels compared to DON alone. In contrast, the combination of DON with DHZEN significantly reduced LY permeability (1.2% ± 0.2%) compared to DON alone (Fig. 7). A decrease in permeability was also observed for GEN + DON (0.9% ± 0.1%) and DHZEN + GEN + DON (1.2% ± 0.2%) compared to DON alone. The impact of EQ (1.6% ± 0.2%) on reducing LY permeability was relatively small compared to the effects caused by GEN (0.9% ± 0.1%), although a slight decrease was observed.

### Viability assay of single substances and combinations

**Fig. 8:**
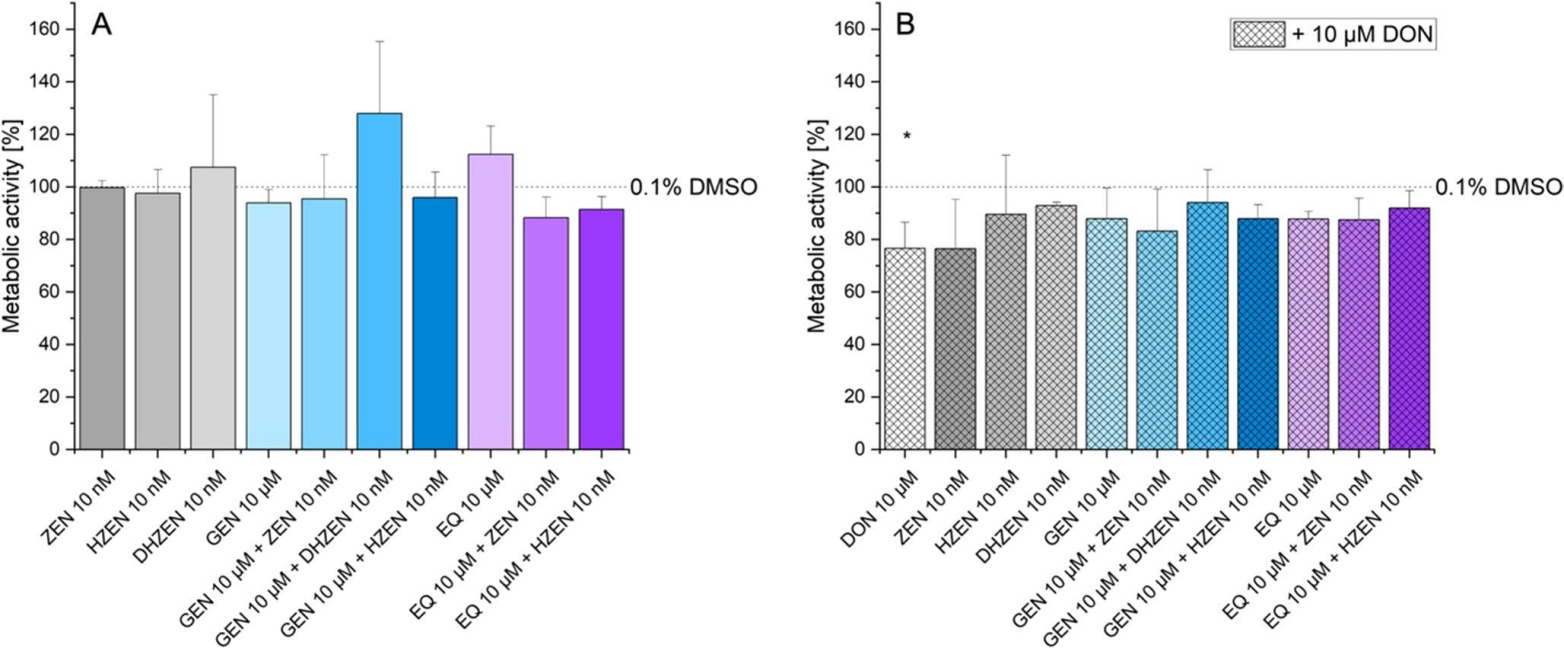
Metabolic activity after 72 h incubation with deoxynivalenol (DON), zearalenone (ZEN), hydrolyzed ZEN (HZEN), decarboxylated hydrolyzed ZEN (DHZEN), genistein (GEN), GEN + ZEN, GEN + HZEN, GEN + DHZEN, R,S-equol (EQ), EQ + ZEN, EQ + HZEN ± 10 µM DON or solvent control (0.1% DMSO). Results are depicted as mean + standard deviation of 4 biological replicates. Significant differences between the solvent control and the incubation conditions were calculated by one-sample Student’s t-test and indicated with “*” (*p* < 0.05), “**” (*p* < 0.01) or “***” (*p* < 0.001)

As shown in Fig. 8A, none of the tested substances led to a significant decrease in metabolic activity compared to the solvent control. Interestingly, DHZEN, GEN + DHZEN, and EQ displayed a trend towards increasing metabolic activity after 72 h. In contrast, co-incubation of these substances with 10 µM DON (Fig. 8B) showed a tendency to reduce metabolic activity compared to the solvent control. However, only the reduction caused by 10 µM DON alone was statistically significant when compared to the solvent control.

### Apical and basolateral measurements for transport assessment

We performed high performance liquid chromatography coupled to tandem mass spectrometry (HPLC-MS/MS) measurements to gain deeper insights into the metabolism of the compounds and their mutual effects. Therefore, we optimized a method for analyzing both ISFs and ZEN along with their metabolites in the apical and basolateral TEER compartments. However, since we applied ZEN and its metabolites in a 1:1000 times lower concentration, it was only possible to detect ISFs and their corresponding metabolites due to the sensitivity of the method.

**Fig. 9:**
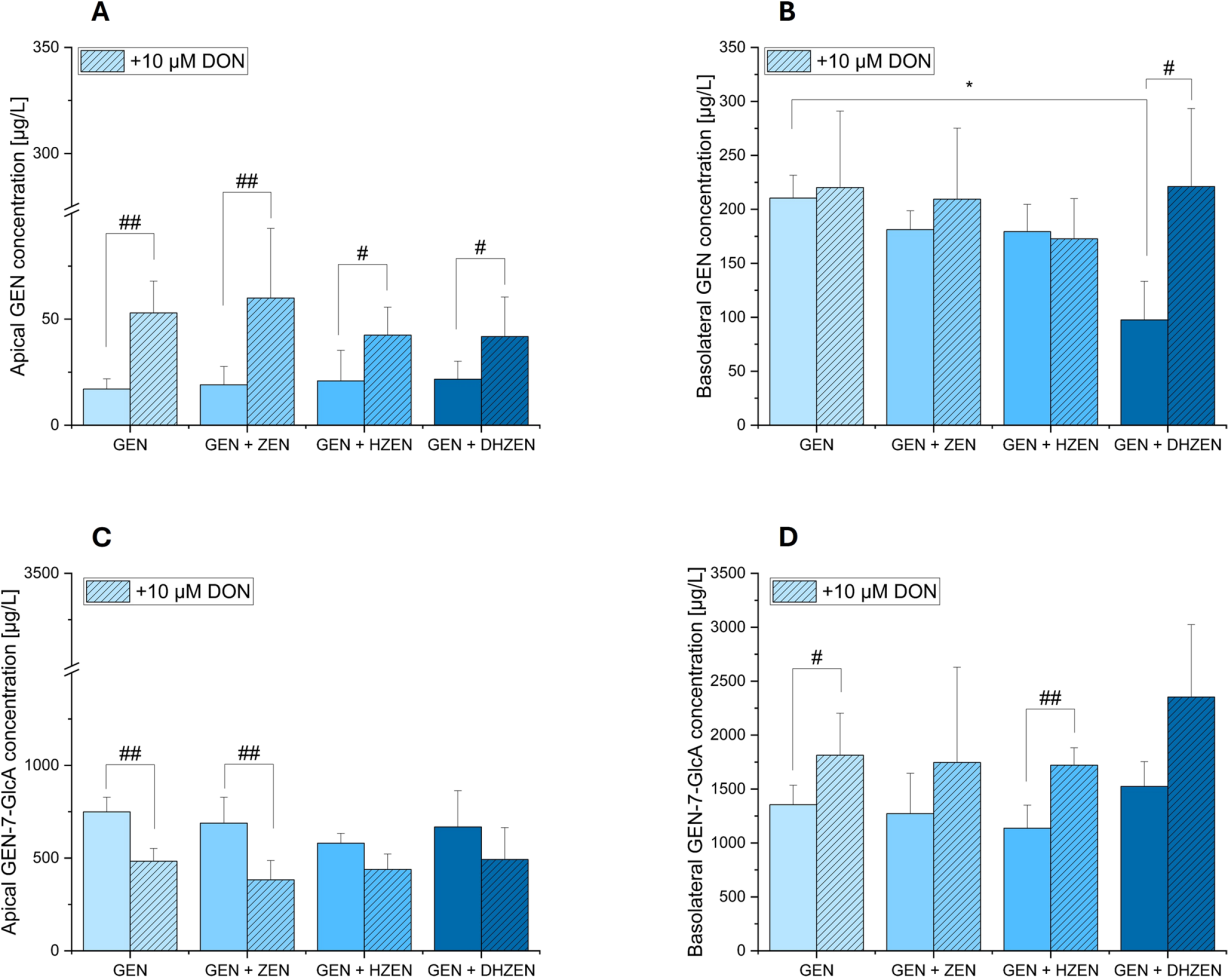
Concentration of genistein (GEN) and genistein-7-glucuronic acid (GEN-7-GlcA) in µg/L of different treatments of IPEC-J2 cells in (**A**) the apical ± 10 µM deoxynivalenol (DON) and (**B**) basolateral ± 10 µM DON compartment of 4 biological replicates. Significant differences between samples were calculated by one-way analysis of variance (ANOVA) and indicated with “*” (*p* < 0.05), “**” (*p* < 0.01) or “***” (*p* < 0.001). Significant differences between samples ± 10 µM DON were calculated by two-sample Student’s *t*-test and indicated with “#” (*p* < 0.05), “##” (*p* < 0.01) or “###” (*p* < 0.001)

**Fig. 10:**
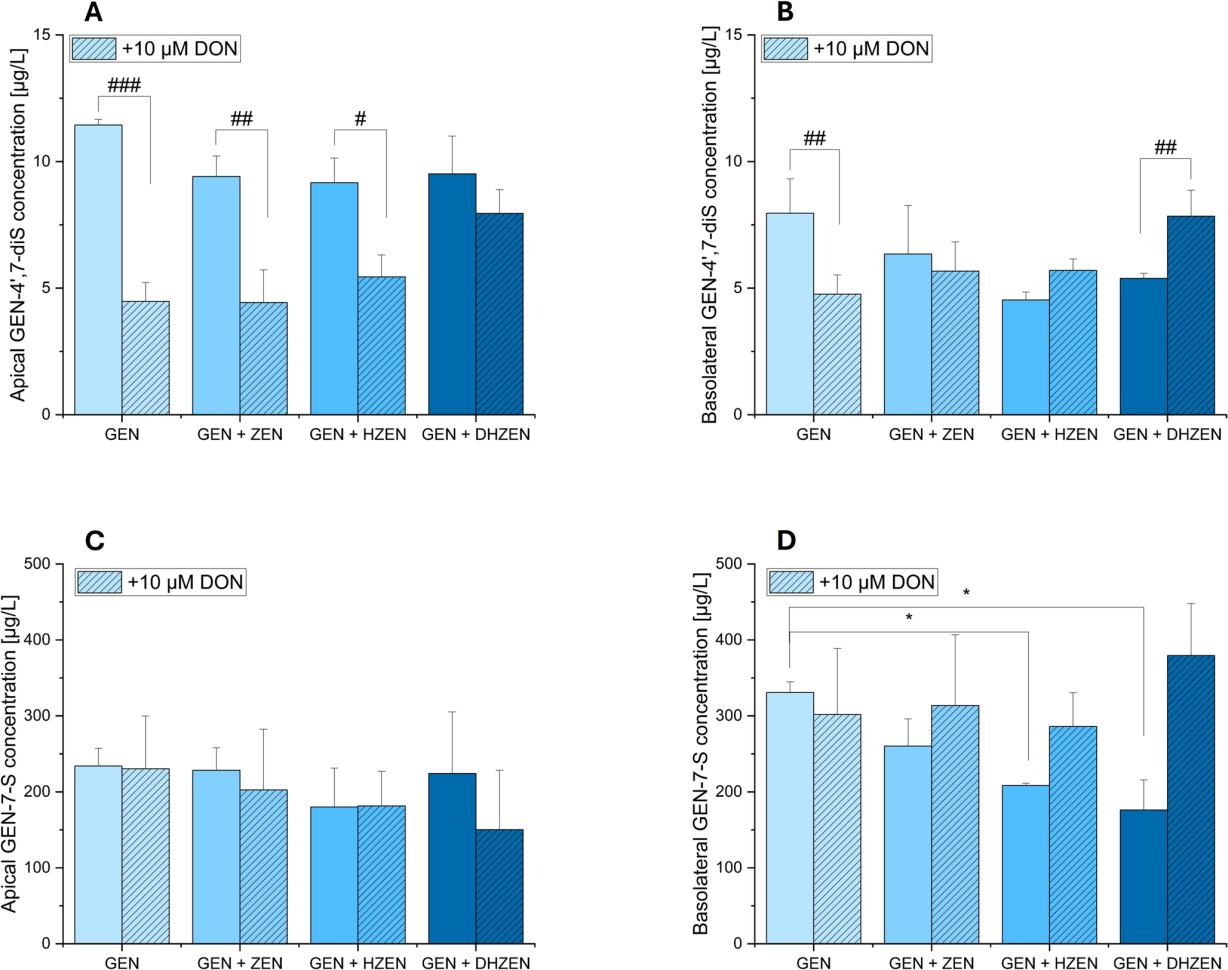
Concentration of genistein-4’,7-di-sulfate (GEN-4’,7-diS) and genistein-7-sulfate (GEN-7-S) in µg/L of different treatments of IPEC-J2 cells in (**A**) the apical ± 10 µM deoxynivalenol (DON) and (**B**) basolateral ± 10 µM DON compartment of 4 biological replicates. Significant differences between samples were calculated by one-way analysis of variance (ANOVA) and indicated with “*” (*p* < 0.05), “**” (*p* < 0.01) or “***” (*p* < 0.001). Significant differences between samples ± 10 µM DON were calculated by two-sample Student’s *t*-test and indicated with “#” (*p* < 0.05), “##” (*p* < 0.01) or “###” (*p* < 0.001)

In our analysis, we identified GEN and its metabolites 4’,7-di-sulfate (GEN-4’,7-di-S), 7-sulfate (GEN-7-S), and 7-glucuronic acid (GEN-7-GlcA) in both the apical and basolateral compartments. The concentration of GEN and its respective metabolites are presented in the following Figs. (9 and 10). Please note that the data for EQ and several of its phase II metabolites are included in the supplements.

In our analysis of GEN metabolites, specifically GEN-4’,7-di-S and 7-GlcA, no significant changes were observed in their concentrations across various cell treatments in both compartments (as shown in Figs. 9 and 10). However, when GEN was co-treated with DHZEN, a significant decrease in GEN concentration was evident in the basolateral compartment, setting it apart from the other treatments (shown in Fig. 9B). In parallel, a slightly higher concentration of GEN-7-GlcA was seen in the same combination in the basolateral compartment (Fig. 9D).

Additionally, combinations involving GEN with HZEN or DHZEN also resulted in lower concentrations of GEN-7-sulfate (GEN-7-S) (Fig. 10) compared to alternative treatments. These findings highlight the complex cellular interactions and metabolic transformations, providing insight into the specific impact of these combinations on GEN metabolite profile.

## Discussion

Individuals are commonly exposed to a wide range of undesirable and toxic compounds simultaneously. Consequently, toxicity assessments should consider not only the effect of single harmful compounds but also their potential interactions with other toxins or bioactive constituents, in order to ensure a comprehensive and reliable risk assessment. As recently reviewed, mycotoxins and phytoestrogens frequently co-occur in various feed commodities (Grgic et al. 2021; Penagos-Tabares et al. 2021, 2023). Therefore, the present study investigates the effects of GEN and the gut microbial metabolite of DAI, EQ, as well as the *Fusarium* toxins ZEN and DON, on intestinal barrier integrity using IPEC-J2 cells.

In regards of the intestine, ZEN has been shown to induce cytotoxic and apoptotic effects on human enterocytes (Abid-Essefi et al. 2003; Kouadio et al. 2005). Furthermore, it disrupts gut barrier integrity by damaging intestinal villi and glands. This is a consequence of intestinal inflammation through upregulation of proinflammatory cytokines and activation of NLRP3 inflammasome, while reducing anti-inflammatory signals like IL-10 (Han et al. 2022). However, its specific impact on the intestinal barrier remains mainly underexplored. Recent studies indicate that ZEN does not significantly affect TEER values in IPEC-1 cells, while its metabolites α-ZEL and β-zearalenol (β-ZEL) lead to a substantial, time-dependent decrease in TEER levels. Furthermore, ZEN exposure in rats has been associated with decreased mRNA expression of claudin-4 (CLD-4) and occluding (Occ) in the jejunum (Marin et al. 2015). CLD-4 and Occ are essential proteins in tight junctions and are critical for maintaining the integrity and function of epithelial or endothelial barrier models in vitro. CLD-4 regulates paracellular permeability by controlling ion selectivity, enhancing the barrier’s ability to restrict ion movement. Together, these proteins help replicate physiological barrier properties, making them crucial for studies on toxin permeability and disease modeling (Liu et al. 2014). In the applied TEER model, no significant impact on the barrier integrity was seen with ZEN and its metabolites, HZEN and DHZEN, after 24, 48, and 72 h of incubation (Fig. 2). Furthermore, LY experiments were conducted, which are a robust indicator of membrane integrity and permeability. LY is a non-interacting fluorescent molecule and serves as a quantitative detector of cell membrane permeability. Figure 7 presents LY permeability measurements for respective substances after 72 h of incubation, providing additional insights into the membrane integrity of IPEC-J2 cells. Neither ZEN nor its degradation products HZEN and DHZEN (all 10 nM), nor the ISFs (both 10 µM), had a significant impact on LY permeability compared to the solvent control (0.1% DMSO). These results are consistent with the TEER results. Similarly, incubation with ISFs (GEN and EQ) did not affect the TEER values or LY permeability compared to the solvent control (0.1% DMSO) (Figs. 3 and 7). Interestingly, when ZEN and its degradation products and ISFs were combined, there was a subtle increase in TEER values after 24 h. However, this effect was not sustained at 48 and 72 h (Fig. 4A).

Exposure to 10 µM DON significantly reduced TEER values to 63% (± 5%) after 24 h, further decreasing to 51% (± 7%) and 49% (± 9%) after 48 and 72 h, respectively (Fig. 2A). Additionally, the combination of all respective substances with 10 µM DON led to an increase in LY permeability, suggesting a disruption in the cell membrane integrity (Fig. 7, bars highlighted in pattern). This is in accordance with previous studies investigating the impact of DON on barrier integrity in IPEC-J2 and Caco-2 cells (Akbari et al. 2014; Beisl et al. 2020; Kadota et al. 2013; Sergent et al. 2006; Springler et al. 2016). DON is known to disrupt the integrity of the barrier by affecting the structure and function of tight junctions. It activates signaling pathways like mitogen activated protein kinase (MAPK), nuclear factor-kappa B (NF-κB), and p38, all involved in modulation of tight junction proteins (Maresca et al. 2008). Moreover, DON increased the phosphorylation of proteins involved in tight junction regulation, leading to their degradation and disassembly. This disruption led to intestinal permeability and inflammation (Maresca 2013).

DON combined with ZEN or HZEN did not lead to a further reduction in TEER values or increase in LY permeability compared to DON alone. In contrast, co-incubation of DON with DHZEN showed a clear mitigating effect in both barrier integrity assays compared to DON alone (Figs. 2B and 7). Specifically, DHZEN seemed to attenuate the DON-induced decline in TEER values, suggesting a potential protective effect. The mechanism underlying the protective activity of DHZEN remains to be fully elucidated. The bioconversion of ZEN to DHZEN presents a plausible hypothesis for the observed phenomenon. ZEN-degrading enzymes, such as the ZEN-lactonase Zhd101p from *Gliocladium roseum* NRRL 1859 and *Clonostachys rosea*, are known to convert ZEN into the non-toxic and non-estrogenic metabolites HZEN and DHZEN (Fruhauf et al. 2019). This transformation results in the introduction of additional hydroxy groups, which may confer antioxidant properties and thereby exert anti-inflammatory effects. Beyond its potential to protect epithelial barrier integrity, the other reported biological activities are its antiestrogenic activity in MCF-7 cells and a yeast estrogen reporter assay. Additionally, DHZEN did not lead to any clinical signs or change in gene expression in a piglet feeding trial compared to the group fed a ZEN diet (Fruhauf et al. 2019). Moreover, co-incubation of ISFs with ZEN or HZEN along with 10 µM DON also counteracted the DON-induced reduction in TEER values (Fig. 4B and Fig. 5B).

Similarly, the combinations of DON with GEN and DON together with GEN and DHZEN also led to a decreased permeability compared to DON alone. This protective effect of ISFs aligns with previous studies demonstrating the barrier-stabilizing properties of ISFs, particularly in intestinal epithelial models. GEN and DAI have been shown to enhance tight junction formation and suppress pro-inflammatory cytokines, thereby supporting intestinal epithelial barrier integrity (Noda et al. 2012). GEN, for instance, increased the expression of proteins essential for tight junction formation, such as Occ and CLD-4, while downregulating the expression of pro-inflammatory cytokines like tumor necrosis factor (TNF) and interleukin-6 (IL-6) (Noda et al. 2012; Sergent et al. 2010). Furthermore, ISFs are known to inhibit inflammation in human intestinal cells, maintaining the integrity of tight junctions, and suppressed the synthesis of inflammatory proteins such as interleukin 8 (IL-8) (Di Cagno et al. 2010). A protective effect of GEN by a TNFα-dependent decrease in TEER values in Caco-2 cells was already reported (Piegholdt et al. 2014). TNF-α impairs barrier function by reducing tight junction and adherent junction protein levels, promoting internalization of junctional proteins, and inducing cytoskeletal contraction. Interestingly, GEN did not alter the protein levels of junction proteins but reduced the concentration of soluble tumor necrosis factor-1 (sTNFR1), reduced disintegrin and metalloprotease 17 (ADAM17) mediated shedding of TNFR1. Additionally, GEN reduced NF-κB transactivation induced by TNF-α, suggesting their potential in inhibiting inflammation-driven barrier loss. GEN reduced the phosphorylation of zonula occludens-1 (ZO-1) tyrosine residues, a critical protein in tight junction complexes. Phosphorylation of this proteins impairs intestinal epithelial barrier function (Piegholdt et al. 2014). Furthermore, GEN treatment at various concentrations has been associated with reduced bacterial internalization in Caco-2 cells, accompanied by increased TEER values. This enhancement of barrier integrity appeared to counteract the TEER decrease induced by bacterial contamination (Wells et al. 1999).

It is well known that ISFs are mainly metabolized via phase II reactions in the intestine and liver, resulting in glucuronide (GlcA) and sulfate conjugates that circulate in the blood stream. In Caco-2 cells, a clear preference for glucuronidation, specifically at the 7-hydroxy group, was observed over sulfation at the 4’-hydroxy group (Yuan et al. 2012). When ISFs have both 7 and 4’-hydroxy groups available (such as GEN), the predominant excretion products are glucuronides. These findings demonstrate a consistent tendency for glucuronidation over sulfation in humans (Chen et al. 2005). Therefore, we analyzed the GEN metabolites GEN-4’,7-di-S, GEN-7-S, and GEN-7-GlcA in the apical and basolateral compartments, representing the luminal and abluminal side of the intestine. As illustrated in Figs. 9 and 10, these results provide insights into the complex interactions and metabolism of these substances within the cellular environment. Upon examining GEN metabolites, specifically GEN-4’,7-di-S and GEN-7-GlcA, in both compartments (as shown in Fig. 10), remained stable across all treatment conditions, indicating that GEN metabolism was not significantly altered by co-incubated compounds.

Interestingly, co-treatment of GEN with DHZEN resulted in a remarkable decrease in GEN concentration in the basolateral compartment, distinguishing this from all other treatments (as depicted in Fig. 9B). This reduction may be linked to the observed trends towards higher level of GEN-7-GlcA in the same treatment group. Although other GlcA and sulfate metabolites were included in the analytical method, they were not detected under the conditions applied. These findings imply that the potential protective effect of GEN observed in the TEER experiments, which was more evident when administered alone with DON compared to the combination of DON with GEN and DHZEN, are primarily mediated by GEN itself rather than its metabolites. The decrease in GEN concentration following co-incubation with DHZEN highlights the specificity and complexity of metabolic interactions between these compounds. Furthermore, combinations of GEN with either HZEN or DHZEN resulted in lower concentrations of GEN-7-S compared to other treatment groups (Fig. 10D).

In conclusion, this study comprehensively evaluated the effects of various compounds on the integrity of cellular barriers. Specifically, both ZEN and its metabolites, HZEN and DHZEN, as well as ISFs GEN and EQ, did not significantly impair barrier integrity after 24, 48, and 72 h of incubation. In contrast, exposure to 10 µM DON significantly reduced barrier integrity, indicating disruption of tight junction structure and function, which are key elements in maintaining intestinal epithelial barrier. DON is known to activate signaling pathways like MAPK, NF-κB, and p38, which contribute to the degradation and disassembly of proteins involved in tight junction regulation, escalating intestinal permeability and inflammation (Maresca 2013; Maresca et al. 2008).

Co-incubation of DON with ZEN or HZEN did not significantly alter barrier integrity. However, co-incubation of DON with DHZEN resulted in a milder impairment on barrier integrity compared to DON alone, suggesting a potential protective effect of DHZEN. Moreover, combinations of GEN with HZEN or DHZEN mitigated the DON-induced reduction in barrier function. These protective effects are consistent with previous studies indicating the barrier-stabilizing properties of ISFs, which enhance tight junction formation and suppress pro-inflammatory cytokines, thereby preserving intestinal epithelial barrier integrity.

Furthermore, our analysis of GEN metabolites provided valuable insights into their metabolic pathways within our experimental model. GEN and its major metabolites, particularly GEN-4’,7-di-S and GEN-7-GlcA, remained stable in concentration across all cell treatments. However, a unique trend emerged when GEN was co-treated with DHZEN, leading to a decrease in GEN concentration in the basolateral compartment.

Given the frequent prevalence and co-occurrence of ISFs with mycotoxins in the diet, it is essential to assess their combined toxicological effects than evaluating individual compounds. The present study highlights the complex interactions between mycotoxins such as DON, ZEN, and its degradation products, and ISFs in the context of epithelial barrier integrity and function. The findings reflect a more realistic exposure scenario and emphasize the importance of considering mixture effects in risk assessment.

## Supplementary Information

Below is the link to the electronic supplementary material.

Supplementary File 1 (PDF 548 KB)

## Data Availability

The authors declare that the data supporting the findings of this study are available within the paper and its Supplementary Information files. Should any raw data files be needed in another format they are available from the corresponding author upon reasonable request.

## Abbreviations

CLD-4: claudin-4
DAI: daidzein
DHZEN: decarboxylated hydrolyzed zearalenone
DMEM: Dulbecco’s modified eagle medium
DON: deoxynivalenol
E4’S: (R,S)-equol-4’-sulfate
E7G: (R,S)-equol-7-glucuronide
EQ: R,S-equol
G4’,7dG: genistein-4’,7-diglucuronide
G4’,7dS: genistein-4’,7-disulfate
G4’G: genistein-4’-glucuronide
G4’G7S: genistein-4’-glucuronide-7-sulfate
G7G: genistein-7-glucuronide
G7G4’S: genistein-7-glucuronide-4’-sulfate
G7S: genistein-7-sulfate
GEN: genistein
HPLC-MS/MS: High performance liquid chromatography coupled to tandem mass spectrometry
HZEN: hydrolyzed zearalenone
ISFs: isoflavones
LY: lucifer yellow
NR: neutral red
PBS: phosphate-buffered saline
TEER: transepithelial electrical resistance
TJ: tight junctions
ZEN: zearalenone
α-ZEL: α-zearalenol
