# Supplementary material for: Modulatory effects of isoflavones and zearalenone metabolites on deoxynivalenol induced gut barrier toxicity in vitro
Source: Mycotoxin Res. 2026 Mar 30;42(2):36. doi: 10.1007/s12550-026-00645-1 (PMC13035645; doi:10.1007/s12550-026-00645-1)
Supplement: Supplementary file 1 — (PDF 548 KB) [file 12550_2026_645_MOESM1_ESM.pdf]

Supplementary information of

Modulatory effects of isoflavones and zearalenone metabolites on deoxynivalenol induced gut barrier toxicity *in vitro*

Dino Grgic[1,2], Barbara Novak[3], Elisabeth Varga[1,4*], Doris Marko[1]

1 Department of Food Chemistry and Toxicology, Faculty of Chemistry, University of Vienna, Währinger Str. 38-40, 1090 Vienna, Austria

2 University of Vienna, Doctoral School in Chemistry, Währinger Str. 38-42, 1090 Vienna, Austria

3 dsm-firmenich, Animal Nutrition & Health R&D Center, Technopark 1, 3430 Tulln, Austria

4 Food Hygiene and Technology, Centre for Food Science, Clinical Department for Farm Animals and Food System Transformation, University of Veterinary Medicine, Vienna, Veterinärplatz 1, 1210 Vienna, Austria

* corresponding author: elisabeth.varga@vetmeduni.ac.at

ORCID ID: Dino Grgic: 0000-0001-8753-7027; Barbara Novak: 0000-0002-6200-7836; Elisabeth Varga: 0000-0001-6046-3259; Doris Marko: 0000-0001-6568-2944

<u>*Results for equol (EQ) and its metabolites measured by high performance liquid chromatography coupled to tandem mass spectrometry (HPLC-MS/MS)*</u>

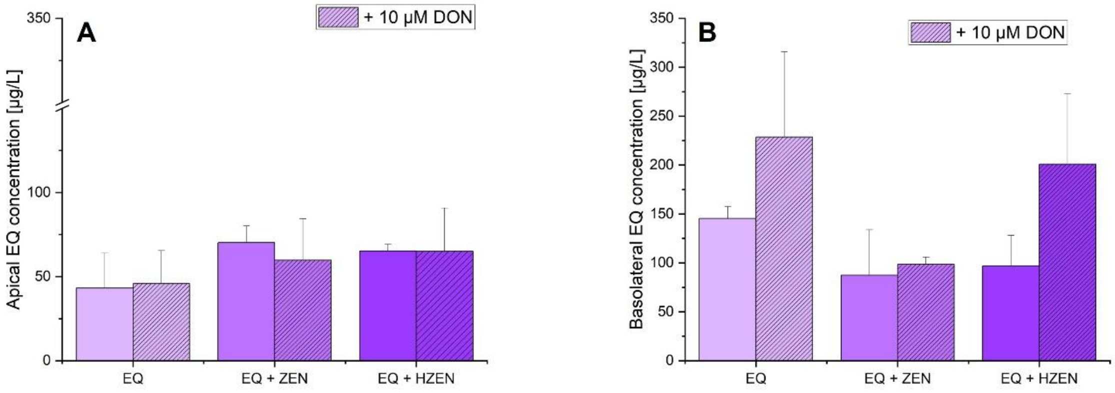

SI Figure 1: Concentration of equol (EQ) in µg/L of different treatments of IPEC-J2 cells in A) the apical ± 10 µM deoxynivalenol (DON) and B) basolateral ± 10 µM DON compartment of 4 biological replicates.

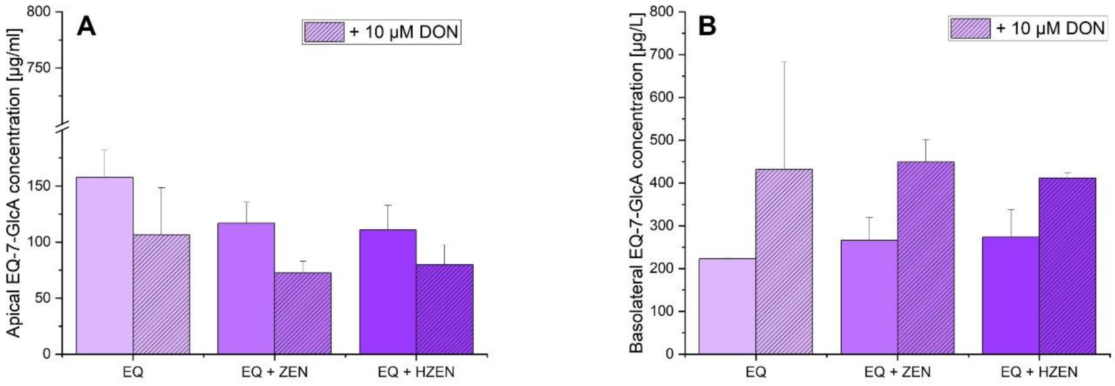

SI Figure 2: Concentration of equol-7-glucuronide (EQ-7-GlcA) in µg/L of different treatments of IPEC-J2 cells in A) the apical ± 10 µM deoxynivalenol (DON) and B) basolateral ± 10 µM DON compartment of 4 biological replicates).

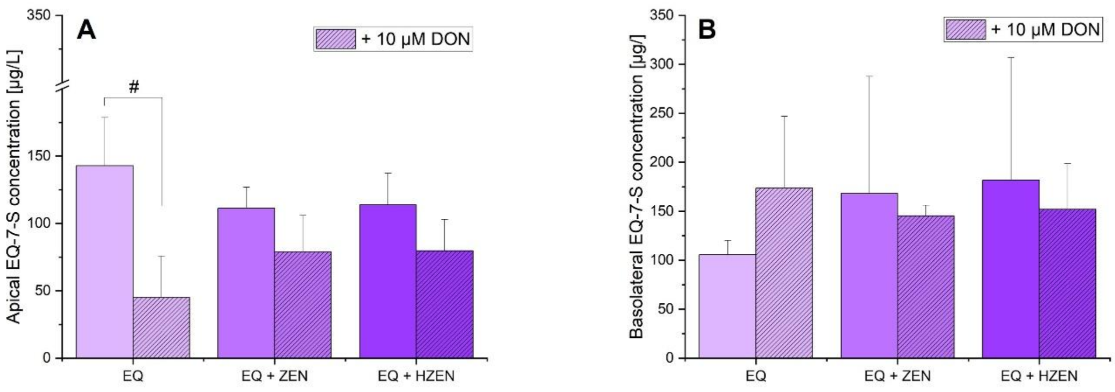

SI Figure 3: Concentration of equol-7-sulfat (EQ-7-S) in µg/L of different treatments of IPEC-J2 cells in A) the apical ± 10 µM deoxynivalenol (DON) and B) basolateral ± 10 µM DON compartment of 4 biological replicates. Significant differences between samples ± 10 µM DON were calculated by two-sample Student's t-test and indicated with "#" ($p < 0.05$), "##" ($p < 0.01$) or "###" ($p < 0.001$).

Supplementary Table S1: Multiple reaction monitoring parameters of the UHPLC-MS/MS method.

Compound Name	Q1 (*m/z*)	DP (V)	RT (min)	Q3 (*m/z*)	Ratio [*)]	CE (V)	CXP (V)
GEN	268.9	-45	11.1	133.1	-	-44	-15
				159.0	0.42	-42	-21
				131.8	0.36	-48	-11
GEN-4'-glucuronide	444.9	-60	7.8	268.9	-	-34	-17
				113.0	0.39	-22	- 7
				175.0	0.22	-20	-13
GEN-7-glucuronide	444.9	-60	7.2	268.9	-	-34	-17
				113.0	0.52	-22	- 7
				175.0	0.40	-20	-13
GEN-4',7-diglucuronide	620.8	-105	4.5	268.9	-	-56	-15
				444.8	0.88	-28	-29
				112.9	0.27	-30	-17
GEN-7-sulfate	348.8	-15	9.1	269.0	-	-28	-21
				132.9	0.08	-56	-19
				131.9	0.04	-68	-13
GEN-4',7-disulfate	428.8	-40	7.5	268.9	-	-36	-17
				348.9	0.69	-22	-23
GEN-7-glucuronide-4'-sulfate	524.8	-20	5.8	269.1	-	-50	-17
				348.7	0.43	-36	-23
				112.9	0.04	-34	-11
GEN-4'-glucuronide-7-sulfate	524.8	-20	6.3	269.1	-	-50	-17
				445.0	0.66	-28	- 3
				112.9	0.10	-34	-11
DAI	252.8	-95	10.2	132.0	-	-50	-13
				223.8	0.99	-36	-15
				208.0	0.97	-42	-13
DAI-4'-glucuronide	428.9	-40	7.1	253.0	-	-34	-17
				112.9	0.59	-20	-15
				174.9	0.33	-18	-15

Supplementary Table S1: continued

Compound Name	Q1 (*m/z*)	DP (V)	RT (min)	Q3 (*m/z*)	Ratio [*)]	CE (V)	CXP (V)
DAI-7-glucuronide	428.9	-40	6.4	253.0	-	-34	-17
				112.9	0.69	-20	-15
				174.9	0.62	-18	-15
DAI-4',7-diglucuronide	604.8	-20	3.5	253.0	-	-56	-15
				429.0	0.72	-26	-25
				112.9	0.40	-28	- 7
DAI-4'-sulfate	332.8	-20	8.5 [1)]	253.0	-	-30	-21
				116.9	0.22	-52	-19
				134.8	0.06	-44	- 9
DAI-4',7-disulfate	412.8	-30	6.9	253.1	-	-38	-19
				332.8	0.91	-18	-23
				224.9	0.08	-64	-19
DAI-7-glucuronide-4'-sulfate	508.8	-30	5.2	253.0	-	-52	-17
				332.9	0.65	-34	-21
				174.7	0.01	-42	-15
DAI-4'-glucuronide-7-sulfate	508.8	-30	5.7	253.0	-	-52	-17
				429.0	0.62	-30	-29
				174.7	0.03	-42	-15
EQ	240.9	-80	10.8	121.1	-	-20	- 9
				118.9	0.72	-26	-13
				134.9	0.47	-24	-19
EQ-7-glucuronide	416.9	-70	7.7	113.0	-	-26	-19
				174.9	0.42	-22	-13
				120.9	0.27	-40	-17
EQ-4'-sulfate	320.8	-60	8.9 [2)]	121.1	-	-36	-21
				119.0	0.79	-40	-17
				241.0	0.68	-28	-15

The entrance potential (EP) was set to -10 V for all transitions.

Abbreviations: GEN – genistein, DAI – daidzein, EQ – S-equol, Q1 – precursor mass, DP – declustering potential, Q3 – fragment mass, CE – collision energy, CXP – cell exit potential, RT – retention time.

*) The ratio always refers to the first provided transition for each metabolite which was also the one with the highest intensity.

1) The obtained standard does not seem to be as pure as stated since a double peak was visible. Since no baseline separation was achieved and both compounds were present in the standard the sum of the peak areas was taken into consideration for all the evaluations.

2) No standard for EQ-7-sulfate was available, no clear second peak was visible in the samples, but both isoforms might be present.
